# Fear vs. temptation: a push-pull-mooring perspective on users' continuance intention toward AI face-swapping technology

**DOI:** 10.3389/fpsyg.2026.1781164

**Published:** 2026-04-10

**Authors:** Yanqing Xia, Yi Rong, Yu Shao, Yang Wang

**Affiliations:** 1 School of Design, NingboTech University, Ningbo, China; 2 School of Art, Sungkyunkwan University, Jongno-gu, Seoul, Republic of Korea; 3 Academy of Fine Arts, Guangdong University of Education, Guangzhou, China; 4 Department of Digital Media, School of Humanities and Arts, Hunan International Economics University, Changsha, Hunan, China

**Keywords:** AI face-swapping technology, continuance intention, digital literacy, push-pull-mooring model, self-efficacy, technology anxiety

## Abstract

The explosive growth of AI face-swapping technology has created a “technology paradox” where users experience simultaneous attraction and apprehension. To decode the underlying mechanisms of this paradox, this study utilizes the Push-Pull-Mooring (PPM) framework to construct an integrative model. We examine users' continuance intention by incorporating push effects (fear-driven), pull effects (temptation-driven), and individual mooring factors (digital literacy and self-efficacy). Data were collected from 351 active users of FacePlay in China and analyzed using Partial Least Squares Structural Equation Modeling (PLS-SEM). The results indicate that while both push and pull effects significantly influence continuance intention, the pull effect (temptation) exerts a substantially more dominant impact. Digital literacy and self-efficacy not only directly enhance continuance intention but also function as a dual-moderating mechanism—simultaneously buffering the inhibitory influence of push effects and amplifying the facilitating influence of pull effects (temptation) on continuance intention. This study offers novel theoretical insights into the technology paradox and provides concrete implications for digital literacy education and risk governance.

## 1 Introduction

In recent years, AI face-swapping technology has leveraged deep learning algorithms to transfer one individual's facial features onto another through facial recognition, feature extraction, and facial fusion operations, achieving highly realistic visual effects with broad applications in video and image processing (Syed Abu Bakar et al., 2026; Nguyen et al., 2025). China possesses one of the world's most advanced short-video industries (Xie et al., 2019), and industry data indicate that the number of users of AI face-swapping applications in China exceeded 200 million in 2023, with representative platforms such as FaceApp and Reface accumulating massive active user bases on mobile devices (Juefei-Xu et al., 2022). AI face-swapping technology has rapidly proliferated owing to its low entry barrier, high entertainment value, and strong shareability. However, accompanying its powerful entertainment appeal are increasingly severe risks of identity theft, privacy invasion, and technological misuse (Rehaan et al., 2024; Xie and Duan, 2025). The coexistence of creative temptation and fear of rights violation constitutes a fundamental characteristic that distinguishes AI face-swapping technology from other consumer information technologies.

This paradoxical coexistence of temptation and fear is a structural feature prevalent in the diffusion of emerging technologies. Mick and Fournier (1998) observed in their seminal study on consumer–technology relationships that users interacting with emerging technologies often simultaneously experience emotionally opposing yet coexisting states—a phenomenon termed the “technology paradox.” This framework reveals a critical insight: users do not make binary choices between “use” and “avoidance” but rather arrive at behavioral decisions within the sustained tension between two competing forces. AI face-swapping technology makes this paradox particularly pronounced due to its direct manipulation of the face—the most symbolically significant biometric identifier of individual identity—such that users inevitably perceive a threat to their self-boundary even as they enjoy the entertainment experience. This tension mirrors the cognitive-affective conflict described in dual-process perspectives, wherein affectively driven avoidance responses to fear-inducing stimuli operate concurrently with deliberatively driven approach motivations toward tempting rewards, producing the behavioral ambivalence that characterizes AI face-swapping use decisions.

How, then, do users arrive at continuance decisions under the joint influence of temptation and fear? Existing literature exhibits a pronounced fragmentation of perspectives. Most research focuses primarily on the negative effects of AI face-swapping technology, framing users as passive victims, covering deepfake detection (Tolosana et al., 2020), ethical risks (Okviosa et al., 2025; Yin, 2024; Alanazi et al., 2025), and public trust crises (Nguyen et al., 2025). These studies conclude that individuals struggle to identify deepfake content, and that criminals may exploit face-swapping technology to perpetrate fraud or disseminate disinformation. Other studies focus on users' adoption of AI face-swapping technology, arguing that cognitive factors such as entertainment value, ease of use, and social influence are the key drivers of users' intention to use face-swapping applications (Nguyen et al., 2025; Sivathanu et al., 2022; Ha et al., 2024). Both streams suffer from a fundamental limitation: the former overlooks the powerful positive motivations sustaining continued use, while the latter ignores the emotional conflict that arises when users simultaneously confront privacy threats and entertainment inducements. In other words, existing research either explains “why users adopt” or “why users avoid,” while few studies have examined how continuance intention actually forms when temptation and fear coexist.

The Push-Pull-Mooring model offers a theoretical foundation for addressing this gap. Proposed by Bansal et al. (2005), the Push-Pull-Mooring model structurally accommodates both sources of behavioral resistance (push) and sources of behavioral motivation (pull), while capturing the moderating role of individual-level conditions on behavioral transitions through mooring variables (Bansal et al., 2005). The model has been widely validated across multiple behavioral contexts in information systems research, including user switching behavior, platform migration, and technology substitution (Wang M. et al., 2025; Lisana, 2023; Liang and Wei, 2024). Unlike theoretical frameworks that focus on a single dimension, the Push-Pull-Mooring model is uniquely capable of systematically integrating fear-driven resistance and temptation-driven motivation within a single model, offering a distinctive theoretical advantage for understanding the paradoxical behavioral decisions that characterize AI face-swapping technology use.

The fear associated with AI face-swapping technology stems primarily from its direct manipulation of facial biometric features, generating threat perceptions qualitatively distinct from general privacy risks. These concerns are not a generalized rejection of technology but rather point to three psychologically distinct yet interrelated dimensions. First, identity-level threat perception: AI face-swapping technology renders any individual's face potentially subject to unauthorized manipulation, dissemination, or misuse. Recent research demonstrates that the identity-manipulation properties inherent in deepfake technology generate fears of losing control over one's self-image, intensifying as technological realism improves (Mansoor, 2024)—a threat experience corresponding to a perceived violation of the integrity of one's self-boundary. Second, privacy-level invasion perception: the processes of facial data collection and algorithmic processing are highly opaque, and users widely fear the misuse and leakage of their personal biometric information. Perceived privacy invasion has been repeatedly confirmed as a core driver of user resistance in AI technology contexts (Dahabiyeh et al., 2025; Wang, 2025), with privacy risk perceptions involving facial biometric features carrying an irreversibility that produces psychological impact far exceeding general data privacy concerns (Cowan et al., 2021). Third, trust-level technology distrust: the opacity of underlying algorithms leaves users unable to ascertain how their biometric data are processed or stored, while repeated high-profile misuse cases—including AI face-swap fraud and disinformation incidents—have driven a sustained rise in systemic public distrust (Hriez, 2025; Xu, 2023). Accordingly, this study integrates perceived identity threat, perceived privacy invasion, and technology distrust into a push effect construct, examining the fear-driven forces that “push” users away from AI face-swapping technology.

Nevertheless, the aforementioned fears have not prevented AI face-swapping technology from continuing to proliferate globally, a phenomenon that itself reveals the powerful temptations operating alongside fear. First, entertainment-level immediate gratification: AI face-swapping technology enables users to instantaneously embody any role, and the immediate creative satisfaction of this experience carries high immersiveness. Research indicates that perceived enjoyment is among the strongest predictors of continued use of AI face-swapping technology (Nguyen et al., 2025). Second, utilitarian-level functional value: AI face-swapping technology has transcended purely recreational applications, demonstrating substantial practical value in content creation, brand marketing, and personal image management. Users' perceptions of the functional utility of the technology directly reinforce their continuance intention, with perceived usefulness having been repeatedly validated in the adoption of AI-generated content technologies (Khan and Hasan Emon, 2024; Kim et al., 2025). Third, social identification drive: recent research suggests that users employ AI face-swapping content to construct and perform self-identity (Santo, 2024); given the inherently high transmissibility and topical salience of such content on social platforms, creating and sharing face-swapped material enables users to obtain not only social interaction but also a sense of group belonging and social identification (Kaya, 2025; Ashforth and Mael, 1989; Hogg and Terry, 2000; Turner et al., 1979). This study integrates these three categories of temptation into a pull effect construct, operationalized as perceived enjoyment, perceived usefulness, and need for social identification.

Even when facing identical push and pull forces, behavioral outcomes may differ substantially across users, making individual-level conditions an indispensable moderating factor. In artificial intelligence technology contexts, digital literacy and self-efficacy have been empirically confirmed as critical individual-level conditions shaping how users perceive and respond to technological risks. Users with higher digital literacy can more accurately assess the true risk boundaries of a technology, thereby possessing greater cognitive buffering capacity against the negative influence of push effects (Buijsman et al., 2025). Users with stronger self-efficacy maintain a higher sense of control over technology use and, when confronting equivalent threats, are more inclined to sustain rather than avoid usage (Jokisch et al., 2020). This study proposes a dual-moderating mechanism: these variables act as a “buffer” to weaken the inhibitory influence of fear-driven push effects, while simultaneously serving as an “amplifier” to strengthen the facilitating drive of temptation-led pull effects. Accordingly, this study incorporates digital literacy and self-efficacy as mooring variables within the Push-Pull-Mooring framework, examining their moderating roles in the process through which push and pull effects influence continuance intention.

Based on the foregoing analysis, this study constructs a theoretical framework of “fear vs. temptation dynamics” grounded in the Push-Pull-Mooring model, aimed at addressing three core research questions:

RQ1: How do the push factors of AI face-swapping technology (perceived identity threat, perceived privacy invasion, and technology distrust) influence users' continuance intention?

RQ2: How do the pull factors of AI face-swapping technology (perceived enjoyment, perceived usefulness, and need for social identification) influence users' continuance intention?

RQ3: How do digital literacy and self-efficacy exert a dual-moderating effect—specifically, the buffering effect on the push-intention path and the amplifying effect on the pull-intention path?

To address these questions, this study draws on a sample of 351 active FacePlay users and employs Partial Least Squares Structural Equation Modeling for empirical testing. This study makes three theoretical contributions: first, extending the Push-Pull-Mooring framework to an AI face-swapping technology context under the lens of the technology paradox, providing a systematic explanation of continuance intention formation mechanisms where temptation and fear coexist; second, offering refined conceptualization of the push dimension tailored to the facial biometric intervention characteristics of AI face-swapping technology, distinct from general privacy risk; and third, revealing the dual-moderating mechanism of digital literacy and self-efficacy. By identifying the buffering and amplifying roles of these mooring variables, this study enriches the theoretical understanding of technology paradoxes. The findings also offer evidence-based guidance for platform designers seeking to alleviate user anxiety and optimize risk communication strategies.

## 2 Research theory and hypothesis

### 2.1 Push-pull-mooring model

The Push-Pull-Mooring (PPM) model was proposed by Bansal et al. (2005) and originated in population migration studies, initially developed to explain the causes of population movement (Lee, 1966; Moon, 1995). In recent years, the model has been extensively migrated and applied to consumer behavior, user switching, and information systems adoption (Wang R. et al., 2025; Lisana, 2023; Liang and Wei, 2024). The Push-Pull-Mooring model comprises three core components: push effects refer to the psychological resistance driving users toward resistance or avoidance; pull effects refer to the motivational forces attracting users toward a target behavior; and mooring effects capture the individual-level conditions that influence behavioral transitions (Bansal et al., 2005). The core advantage of the Push-Pull-Mooring model lies in the symmetry of its structure: it can simultaneously accommodate both behavioral resistance and motivational forces within a unified framework, providing an integrative theoretical basis for understanding the paradoxical behavior of users who simultaneously resist yet continue to use emerging technologies.

Within the Push-Pull-Mooring framework, push effects refer to the psychological resistance that drives users toward avoidance or resistance tendencies; push factors include negative elements that drive users away from existing services (Moon, 1995). These factors typically originate from user dissatisfaction, perceived risk, or external environmental pressure. In general technology acceptance contexts, typical push factors include privacy concerns (Wang M. et al., 2025; Im et al., 2008), perceived risk (Cheng et al., 2019), distrust (Jung et al., 2017), dissatisfaction (Jung et al., 2017), and low system quality (Lai and Wang, 2015). In social media switching research, for instance, privacy concerns and information overload serve as important push forces driving users away from platforms (Kong and Park, 2021). This study integrates perceived identity threat, perceived privacy invasion, and technology distrust into a push effect construct. AI face-swapping technology requires users to upload facial data containing biometric features; once misused, this immutable biometric information can cause irreversible damage to personal reputation and financial security (Mansoor, 2024). Regarding perceived privacy invasion, the forced withdrawal of the ZAO application within 48 h of its 2019 launch due to predatory privacy terms powerfully demonstrates the strong inhibitory effect of privacy perception on user behavior (Fallis, 2021). Regarding technology distrust, on one hand, underlying algorithms are opaque, leaving users unable to understand how their biometric data are processed or stored; on the other hand, the technology has repeatedly been implicated in high-profile misuse cases—including AI face-swap fraud and disinformation incidents widely covered by media—sustaining a continuous rise in systemic public distrust (Hriez, 2025; Xu, 2023).

Pull factors refer to the positive attributes that attract users to adopt new services or technologies (Moon, 1995). In information systems research, pull factors typically concern the advantageous characteristics of alternative services, such as superior functionality, higher quality, or greater attractiveness (Bansal et al., 2005). Pull factors discussed in prior literature include attractiveness (Chou et al., 2016), relative usefulness (Hsieh et al., 2012), relative ease of use (Ye and Potter, 2011), social presence (Li et al., 2018), and technological compatibility (Cheng and Jin, 2019). However, this study accounts for the distinctive nature of AI face-swapping technology as an entertainment-oriented technology. AI face-swapping technology provides users with a unique identity-transformation experience; the novelty and creative enjoyment of merging one's own image with celebrities or historical figures constitute strong intrinsic hedonic motivation (Ha et al., 2024). Moreover, AI face-swapping tools substantially lower the barrier to producing high-quality video content, enabling ordinary users to leverage technological advantages to achieve greater dissemination influence on social platforms (Xie and Duan, 2025). Recent research further suggests that users employ AI face-swapping content to construct and express self-identity (Santo, 2024). AI face-swapping content is inherently highly transmissible and topically engaging on social platforms; through creating and sharing such content, users obtain not only social interaction but also a sense of group belonging and social identification (Kaya, 2025). This study incorporates perceived enjoyment, perceived usefulness, and need for social identification as pull factors, collectively constituting the positive driving forces for continuance intention toward AI face-swapping technology.

Mooring effects refer to the personal, social, and environmental factors that influence individual behavioral decisions, which can either facilitate or impede behavioral occurrence (Moon, 1995). Prior research on mooring factors has primarily focused on habit (Cheng and Jin, 2019), switching costs (Lai and Wang, 2015), and personal innovativeness (Vijaya Bhaskar, 2017)—variables that demonstrate good fit for explaining platform migration and service-switching behavior. However, with the comprehensive proliferation of AI technology, the decision-making context facing users has undergone a fundamental transformation: the complexity, opacity, and potential risks of AI technology have rendered individuals' cognitive capabilities and sense of technological control critical conditions influencing behavioral decisions, dimensions that traditional mooring variables are ill-equipped to capture.

In AI technology contexts, digital literacy and self-efficacy have been empirically confirmed as critical individual-level conditions shaping how users perceive and respond to technological risks. Users with higher digital literacy can more accurately assess the true risk boundaries of a technology, thereby possessing greater cognitive buffering capacity against the negative influence of push effects (Buijsman et al., 2025). Users with stronger self-efficacy maintain a higher sense of control over technology use and are more inclined to sustain rather than avoid usage when confronting equivalent threats (Jokisch et al., 2020; Zivi et al., 2025). Existing research broadly characterizes digital literacy as a cognitive shield against perceived technological risk (Getenet et al., 2024) and self-efficacy as a psychological resource that reinforces usage motivation while attenuating avoidance tendencies (Jokisch et al., 2020). However, most studies treat these two variables as main-effect predictors, examining their direct influence on usage intention. In the complex context where push and pull forces coexist, their critical role may not lie in directly driving behavior but rather in functioning as boundary conditions. Accordingly, this study incorporates digital literacy and self-efficacy as mooring variables within the Push-Pull-Mooring framework, examining their moderating roles in the process through which push and pull effects influence continuance intention.

### 2.2 Push effects and continuance intention

AI face-swapping technology's direct manipulation of facial biometric features generates threat perceptions qualitatively distinct from general privacy risks, pointing to three psychologically distinct yet interrelated dimensions: perceived identity threat, perceived privacy invasion, and technology distrust.

Perceived identity threat refers to the psychological state in which an individual perceives that their identity or reputation may be compromised (Branscombe et al., 1999). In digital technology contexts, identity threat is typically associated with the misuse of personal information, identity theft, or the fabrication of false content (Kietzmann et al., 2020). The core functionality of AI face-swapping technology is facial substitution, requiring users to upload facial photographs or videos; these biometric identifiers are unique and immutable, and their misuse can lead to severe consequences (Chesney and Citron, 2019). Deepfake technology can be weaponized to produce non-consensual intimate imagery, misleading political content, or fraudulent financial transactions, inflicting irreparable damage on personal reputation and financial security. Vaccari and Chadwick (2020) found that one of the public's greatest concerns regarding AI face-swapping technology is that facial information may be exploited to fabricate false content that undermines individuals' social image.

In the context of AI face-swapping technology, perceived identity threat represents users' subjective assessment of the risk that their facial information will be misused (Shao et al., 2024). He et al. (2025) demonstrated empirically that perceived identity threat significantly reduces user acceptance of deepfake technology. Xie and Duan (2025) further showed that even when users are interested in the entertainment functions of AI face-swapping technology, perceived identity threat continues to suppress their actual usage behavior. Accordingly, this study posits that perceived identity threat constitutes an important component of the push effect and will negatively influence users' continuance intention.

Perceived privacy invasion refers to the degree to which individuals perceive that their privacy rights have been violated through the unauthorized collection, use, or disclosure of personal information (Smith et al., 1996). Research indicates that when deciding whether to adopt a technology, users weigh perceived benefits against perceived privacy risks; when risks outweigh benefits, users opt to discontinue use (Dinev and Hart, 2006). AI face-swapping applications typically require access to users' photo libraries, camera permissions, and facial data, and the collection of such sensitive information triggers privacy concerns (Yin, 2024). In the present study, perceived privacy invasion specifically refers to the degree to which users are concerned that AI face-swapping technology may collect, process, or leak their facial biometric information.

Technology-related risks—such as the possibility of privacy breaches and the misuse of AI-generated content—commonly influence consumer acceptance through perceived risk mechanisms. Sohn (2024) found in mobile application research that perceived privacy invasion is an important negative predictor of users' adoption intention. In the context of AI face-swapping technology, this effect may be particularly pronounced given that facial data constitutes biometric information of elevated sensitivity (Moussa et al., 2025). Following the 2019 launch of the ZAO application, users mounted strong resistance after discovering that its privacy terms granted ZAO and its affiliates “a global, royalty-free, irrevocable, permanent, sublicensable, and transferable license,” resulting in the application being removed from app stores within 24 h and its terms being forcibly revised. This incident vividly illustrates how perceived privacy invasion can substantially affect users' acceptance of and continuance intention toward AI face-swapping technology. Li et al. (2016) noted that when users perceive a technology platform as excessively collecting personal information or failing to adequately protect privacy, they develop resistance and reduce their usage. Therefore, this study regards perceived privacy invasion as one of the core dimensions of the push effect.

Technology distrust: Trust is defined as one party's willingness to believe that another party will act in a manner favorable to oneself (Mayer et al., 1995). In technology contexts, trust reflects users' beliefs regarding the reliability, security, and benevolence of a technological system (Harrison McKnight et al., 2002). Research demonstrates that trust is a prerequisite for users adopting new technologies, and that its absence substantially impedes technology adoption behavior (Gefen et al., 2003).

In the present study, technology distrust is operationalized as the psychological state in which users lack confidence in the operational logic and usage consequences of AI face-swapping technology. This distrust originates from two distinct yet interrelated sources. First, algorithmic opacity: the underlying algorithms of AI face-swapping technology function as a “black box” for ordinary users, who are unable to ascertain how their facial biometric data are collected, processed, stored, or shared. This opacity directly undermines users' sense of control over the technological system and erodes their trust in it (Xie and Duan, 2025). Second, the cumulative accumulation of technology misuse incidents has provoked widespread ethical controversy and a trust crisis among the public (Hancock and Bailenson, 2021). Media reports frequently document cases in which AI face-swapping technology has been weaponized to produce fabricated videos, perpetrate fraud, or disseminate disinformation, and this sustained negative coverage has significantly eroded public trust in the technology (Paris and Donovan, 2019). Technology distrust not only directly reduces users' adoption intention but also amplifies their perceptions of privacy risks and security threats (Hsiao, 2003). Knowles and Hanson (2018) further demonstrated empirically that technology distrust is a significant determinant of technology adoption decisions. In the context of AI face-swapping technology, when users cannot ascertain how their facial data will be used or whether it may be leaked or misappropriated, they develop pervasive suspicion and resistance toward the technology as a whole. Therefore, this study regards technology distrust as one of the core dimensions of the push effect.

Integrating the foregoing three dimensions, this study defines the push effect as a higher-order construct comprising perceived identity threat, perceived privacy invasion, and technology distrust, reflecting users' overall level of anxiety toward AI face-swapping technology. In accordance with the theoretical logic of the Push-Pull-Mooring model and the findings of prior research (Hsieh et al., 2012), the push effect is expected to exert a negative influence on continuance intention. Accordingly, this study proposes:

**H1: The push effect exerts a negative influence on users' continuance intention toward AI face-swapping technology**.

### 2.3 Pull effects and continuance intention

Within the Push-Pull-Mooring framework, pull effects represent the motivational forces that attract users toward a target behavior (Bansal et al., 2005). Despite the numerous risk controversies surrounding AI face-swapping technology, its continued global proliferation itself reveals powerful usage inducements. These inducements point to three psychologically distinct yet interrelated motivational dimensions: perceived enjoyment, perceived usefulness, and need for social identification.

Perceived enjoyment refers to the pleasure and amusement users experience in the process of using AI face-swapping technology. Enjoyment is defined as the degree of fun intrinsic to using an information system, independent of any performance consequences (Davis et al., 1992). Hedonic experience is a core driver of user adoption of entertainment-oriented technologies (Venkatesh et al., 2012). In the context of AI face-swapping technology, perceived enjoyment may derive from the sense of fun users experience—for instance, through the creative process of generating high-quality or highly realistic imagery and video, as well as the satisfaction of meeting one's own or others' expectations. AI face-swapping technology satisfies users' entertainment needs by providing novel, amusing, and creatively stimulating experiences (Yadlin-Segal and Oppenheim, 2021). Users can transform themselves into beloved celebrities, historical figures, or virtual characters through AI face-swapping; the novelty and delight of this identity transformation are simply unavailable through conventional content creation tools. Ramayah and Ignatius (2005) found in website usage research that perceived enjoyment exerts an even stronger influence on continuance intention than perceived usefulness. Lowry et al. (2012) confirmed in hedonic information systems research that perceived enjoyment significantly strengthens continuance intention by activating users' intrinsic motivation. Nguyen et al. (2025) demonstrated that the entertainment value of AI face-swapping technology is the foremost factor attracting users. Accordingly, this study regards perceived enjoyment as the core dimension of the pull effect that will positively influence users' continuance intention.

Perceived usefulness refers to users' subjective judgment that AI face-swapping technology can improve their content creation efficiency or enable them to achieve specific functional goals (Davis, 1989). In the era of social media, content creation capability has become an important means through which users obtain attention and social capital (Kietzmann et al., 2011). AI face-swapping technology has transcended the boundaries of pure entertainment, demonstrating substantial practical value in brand marketing, film and television production, and personal image management. A substantial body of social media research has found perceived usefulness to be one of the most robust predictors of users' continuance intention (Davis, 1989; Venkatesh et al., 2003); content creation tool perceived usefulness has been shown to significantly influence users' continuance intention. In the context of AI face-swapping technology, perceived usefulness reflects users' perception of the technology's value in facilitating creative expression and enhancing social influence. Accordingly, this study incorporates perceived usefulness as one of the core dimensions of the pull effect.

Need for social identification, as operationalized in this study, refers to the psychological motivation of users to seek social recognition, obtain affirmation from others, and establish a sense of group belonging through creating and sharing AI face-swapping content. Research indicates that need for social identification constitutes a core extrinsic motivational factor influencing technology adoption (Bielig et al., 2025). Social identification is an important antecedent of affective commitment and intrinsic motivation, demonstrating strong explanatory power for users' technology usage motivation (Hwang, 2010). In social media environments, social platforms transform users into what has been described as “approval-seeking machines,” capable of satisfying users' needs for recognition, belonging, and self-worth (Butler, 2024). In the context of AI face-swapping technology, users who create content using this technology and share it on social platforms are able not only to obtain recognition and appreciation from others, but also to demonstrate their technological sensibility and innovativeness, thereby satisfying their need for social identification. The inherently high transmissibility and topical salience of AI face-swapping content on social platforms makes it an ideal vehicle for satisfying this need. Recent research demonstrates that users engage in “performative” identity expression through AI face-swapping technology, using it to explore and construct their self-identity (Santo, 2024). Through creating and sharing face-swapped content, users obtain not only social interaction but also a sense of group belonging and social identification (Kaya, 2025). Social network research has further confirmed that need for social identification significantly and positively influences users' participation behavior and continuance intention (Wang et al., 2022). Venkatesh et al. (2003) similarly found that social influence constitutes an important driver of new technology adoption, further underscoring the central role of social dynamics in shaping sustained usage behavior. Accordingly, this study incorporates need for social identification as a core dimension of the pull effect.

Integrating the foregoing three dimensions, this study defines the pull effect as a higher-order construct comprising perceived enjoyment, perceived usefulness, and need for social identification, reflecting the overall level of attractiveness that AI face-swapping technology holds for users. In accordance with the theoretical logic of the Push-Pull-Mooring model and the findings of prior research, the pull effect is expected to exert a positive influence on continuance intention. Accordingly, this study proposes:

**H2: The pull effect exerts a positive influence on users' continuance intention toward AI face-swapping technology**.

### 2.4 Mooring effects: digital literacy and self-efficacy

Digital literacy is defined as the ability to effectively access, evaluate, use, and create information in digital environments (Eshet, 2004; Zhang, 2025; Zin and Osman, 2026). Digital literacy encompasses not only technical operational skills but also the capacity to understand the risks and benefits of digital technologies, privacy protection awareness, and critical thinking (van Deursen and van Dijk, 2014). The concept of digital literacy was originally introduced by Gilster (1997) to describe the core competencies required in the digital age. As digital technology has evolved, the meaning of digital literacy has continuously expanded—from foundational computer operation skills to encompassing information evaluation, digital security, online collaboration, and multiple other dimensions (Hargittai, 2010; Hristovska, 2023).

In technology adoption research, digital literacy is regarded as an important individual characteristic influencing users' technology usage behavior (Park and Kim, 2014; Karahanna et al., 1999). Users with higher digital literacy typically possess deeper understanding of technology, are better able to leverage technological functions effectively, and are more knowledgeable about how to mitigate technological risks (Hargittai and Hsieh, 2012). Elrayah and Jamil (2023) found that digital literacy significantly influences users' perceptions of and coping strategies for online privacy risks. Caton et al. (2022) noted that users with higher digital literacy exhibit stronger adaptability and more rational decision-making patterns when encountering emerging technologies.

In the context of AI face-swapping technology, the moderating role of digital literacy may manifest in two respects. First, users with higher digital literacy are more knowledgeable about how to protect personal privacy and identify potential risks; consequently, the negative influence of push effects on them is attenuated. Helsper and Eynon (2013) demonstrated that digital literacy can function as a psychological buffer mechanism, enabling users to assess technological risks more rationally. Second, users with higher digital literacy are also better positioned to take full advantage of the creative functions of AI face-swapping technology and to extract its practical value in greater depth (Hargittai and Walejko, 2008), meaning that the positive influence of pull effects on them is amplified. Accordingly, this study proposes:

**H3: Digital literacy exerts a positive influence on users' continuance intention toward AI face-swapping technology**.

Regarding the moderating role of digital literacy: digital literacy alters the strength of push and pull effects on continuance intention by influencing the accuracy of users' perceptions of risks and benefits. With respect to push effects, users with higher digital literacy possess stronger risk identification and assessment capabilities. Research demonstrates that digital literacy is closely associated with risk literacy; individuals with higher literacy are better able to accurately evaluate and comprehend various types of risk information, form more accurate judgments regarding potential threats embedded in information, and thereby reduce judgmental bias (Perrin et al., 2025). Digital literacy not only directly influences behavioral intention but also operates indirectly through risk perception: the higher a user's literacy, the more accurate their risk perception and the stronger their cognitive buffering capacity against negative information (Chen et al., 2024). In the AI face-swapping context, this means that users with higher digital literacy can more accurately assess the true threat boundaries posed by the technology, forming a cognitive buffer against negative reactions such as perceived identity threat, perceived privacy invasion, and technology distrust, thereby weakening the inhibitory effect of push forces on continuance intention.

With respect to pull effects, users with higher digital literacy are better able to accurately identify the true use value of the technology. Research finds that digital literacy positively influences users' self-efficacy and their capacity to evaluate technology (Chen et al., 2024), enabling users to more accurately assess the authenticity and value of information (Sirlin et al., 2021). In technology adoption contexts, digital literacy is closely associated with willingness to accept AI technology and benefit perception (Musyaffi et al., 2024), meaning that users with higher digital literacy make more rational judgments about technological benefits, can distinguish which face-swapping applications possess genuine entertainment or creative value, and thereby strengthen the facilitating effect of pull forces on continuance intention.

**H3a: Digital literacy buffers the relationship between the push effect and continuance intention; that is, the higher the digital literacy, the weaker the negative influence of the push effect**.

**H3b: Digital literacy amplifies the relationship between the pull effect and continuance intention; that is, the higher the digital literacy, the stronger the positive influence of the pull effect**.

Self-efficacy is defined as an individual's belief in their capability to successfully perform a specific task or achieve a specific goal (Bandura, 1997). Self-efficacy theory was originally proposed by Bandura (1977), who argued that self-efficacy is a core psychological mechanism influencing individual motivation, emotion, and behavior. In technology contexts, self-efficacy reflects users' confidence in their ability to effectively use and master a technology (Compeau and Higgins, 1995). The concept of technology self-efficacy was introduced into information systems research by Compeau and Higgins (1995), referring to individuals' judgment of their ability to use computers to complete specific tasks. Self-efficacy theory holds that individuals with high self-efficacy exhibit greater psychological resilience and coping capacity when confronting challenges and threats; they are more inclined to regard difficulties as surmountable challenges rather than insurmountable obstacles (Jerusalem, 2002). In technology adoption research, self-efficacy has been shown to be an important factor influencing users' technology acceptance and continuance intention (Venkatesh et al., 2003). Research finds that self-efficacy not only directly influences technology usage behavior but also indirectly affects technology adoption by moderating anxiety levels (Peral-Peral et al., 2020; Chang et al., 2024). Accordingly, this study proposes:

**H4: Self-efficacy exerts a positive influence on users' continuance intention toward AI face-swapping technology**.

Regarding the moderating role of self-efficacy: self-efficacy influences the strength of push and pull effects on continuance intention by altering how users respond to technological risks and benefits. Within the Push-Pull-Mooring framework, self-efficacy has been shown to function as a mooring variable that moderates the effects of both push and pull forces (Chang et al., 2017). With respect to push effects, users with stronger self-efficacy tend to perceive perceived identity threat, perceived privacy invasion, and technology distrust as manageable challenges rather than insurmountable threats; this sense of control weakens the inhibitory effect of push forces on continuance intention. Research finds that within the Push-Pull-Mooring framework, technology self-efficacy negatively moderates the relationship between switching costs and usage inertia—that is, users with higher self-efficacy are less adversely affected by switching costs, which are analogous to push forces (Lin and Hsieh, 2022). Recent research on AI technology further confirms that self-efficacy exerts a significant buffering effect on the relationship between anxiety and adoption intention, with high self-efficacy individuals maintaining positive adoption intentions even at elevated levels of anxiety (Tailor and Tailor, 2025). Moreover, high self-efficacy users are more inclined to adopt adaptive, problem-focused coping strategies rather than resorting to passive avoidance in response to threats (Yu et al., 2025).

With respect to pull effects, users with stronger self-efficacy believe in their ability to effectively master AI face-swapping technology; this capability belief reinforces the facilitating effects of perceived enjoyment, perceived usefulness, and need for social identification on continuance intention, further amplifying the positive influence of pull forces. Research indicates that self-efficacy indirectly enhances users' technology adoption intention by strengthening perceived usefulness and perceived ease of use. Consistently, in privacy protection contexts, privacy self-efficacy has been shown to moderate the relationship between perceived threat and protective behavior (Hong, 2025). This suggests that in the technology context of AI face-swapping—which combines entertainment value with privacy risk—high self-efficacy users are better positioned to fully experience the positive benefits offered by the technology while maintaining a rational response to its risks.

**H4a: Self-efficacy buffers the relationship between the push effect and continuance intention; that is, the higher the self-efficacy, the weaker the negative influence of the push effect**.

**H4b: Self-efficacy amplifies the relationship between the pull effect and continuance intention; that is, the higher the self-efficacy, the stronger the positive influence of the pull effect**.

### 2.5 Research model

**Figure 1:**
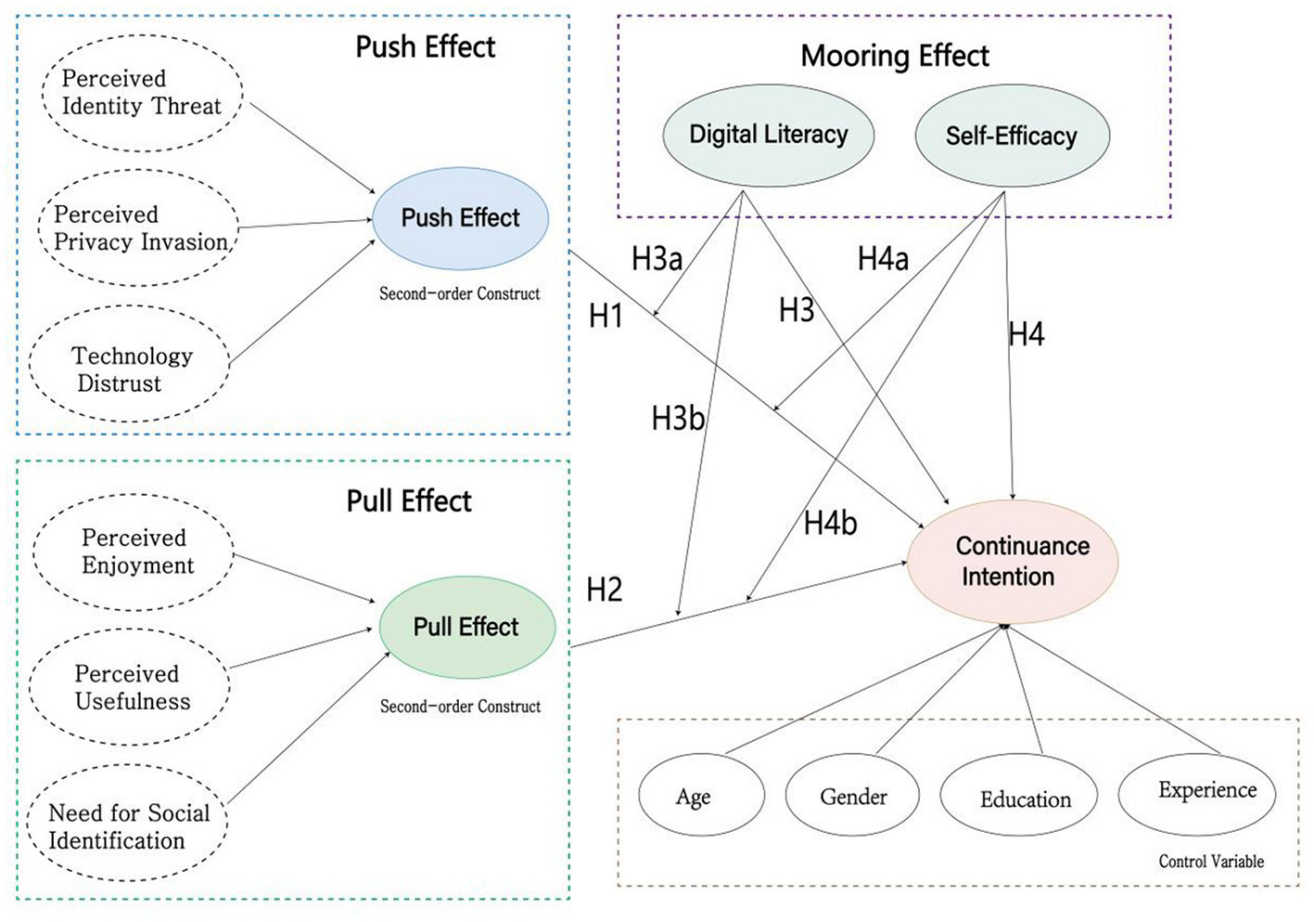
Research model and hypotheses.

Based on the above literature review and hypothesis development, this study constructs the research model shown in Figure 1. Grounded in the PPM theory, this model explores the dynamic equilibrium mechanism of “the game of fear and temptation” in the AI face-swapping technology context. The core logic of the model is as follows:

As shown in Figure 1.

## 3 Research methodology

### 3.1 Questionnaire design and translation

This study employed a questionnaire survey method to collect data. Questionnaire items were primarily adapted from well-established scales published in top-tier international journals to ensure measurement reliability and validity. Since the target respondents were Chinese users, the questionnaire items were first translated into Chinese by two information management professors, then back-translated into English by another translator with specialized English-Chinese translation training. The Chinese translation ensured that respondents could read the questionnaire effortlessly, avoiding measurement errors due to language comprehension barriers. A professional translator performed back-translation to ensure content accuracy, and the results were compared with the original English scales to verify semantic consistency (Brislin, 1970).

The appropriateness of questionnaire items was initially examined by two associate professors and three doctoral students specializing in information systems. These experts possessed in-depth understanding of AI face-swapping technology, technology adoption theories, and questionnaire design methods, and they evaluated and provided modification suggestions regarding theoretical fit, item clarity, and content coverage completeness. Additionally, 42 undergraduate students from one class served as a pilot test sample to examine the face validity and comprehensibility of questionnaire items (Mokkink et al., 2010). Pilot test results indicated that all items demonstrated acceptable comprehensibility, with minor adjustments made to a few ambiguously worded items based on student feedback.

Subsequently, an expert panel consisting of three professors and five doctoral students was invited to complete, modify, and refine the questionnaire draft to ensure the validity and applicability of this study. The expert panel paid particular attention to the fit between questionnaire items and the AI face-swapping technology context, as well as the accuracy of operational definitions for each PPM model construct. Through multiple rounds of discussion and revision, the questionnaire content integrated theoretical constructs with actual usage scenarios of AI face-swapping technology to ensure content validity.

### 3.2 Measurement scales

All constructs in this study were measured using seven-point Likert scales (1 = strongly disagree, 7 = strongly agree) to enhance measurement discrimination and sensitivity (Finstad, 2010). All items were adapted from well-established scales in prior literature and modified to fit the AI face-swapping technology context.

Push effect was operationalized as a second-order construct comprising three sub-dimensions: perceived identity threat (adapted from Vaccari and Chadwick, 2020), perceived privacy invasion (adapted from Xu et al., 2011; Kim and Sung, 2022), and technology distrust (adapted from Harrison McKnight et al., 2002; Söllner et al., 2016).

Pull effect was operationalized as a second-order construct comprising three sub-dimensions: perceived enjoyment (adapted from Venkatesh et al., 2003; van der Heijden, 2004), perceived usefulness (adapted from Davis, 1989; Venkatesh and Davis, 2000), and need for social identification (adapted from Utz et al., 2012).

Mooring factors included digital literacy (adapted from Ng, 2012; Hwang et al., 2023) and self-efficacy (adapted from Compeau and Higgins, 1995; Venkatesh et al., 2003).

Continuance intention was measured using three items adapted from Bhattacherjee (2001) and Cheng (2020).

Control variables included age, gender, education level, and usage experience (measured in months).

A complete list of measurement items and their sources is provided in Table 1.

**Table 1:** Measurement items and operational definitions.

Constructs	Items	Explanations	References
Perceived privacy invasion (PPI)	PPI1 I am concerned that my facial data will be misused by AI face-swapping technology.	The degree to which users perceive that AI face-swapping technology may collect, process, or disclose their facial biometric data in ways that violate their privacy rights.	Xu et al., 2011; Kim and Sung, 2022
	PPI2 AI face-swapping apps collect my facial information without my permission.		
	PPI3 I am concerned that AI face-swapping technology will completely expose my personal portrait and biometric information (such as facial features) to risk.		
Perceived identity threat (PIT)	PIT1 I worry that someone might create fake face-swapped videos of me to damage my reputation.	The degree to which individuals perceive that AI face-swapping technology may manipulate, steal, or abuse their social identity, personal image, or reputation without authorization.	Smith et al., 1996; Vaccari and Chadwick, 2020
	PIT2 I feel uneasy about being unable to distinguish between authentic and fake online content.		
	PIT3 I believe this technology makes everyone's social identity extremely vulnerable to theft and manipulation.		
Technology distrust (TD)	TD1 I do not believe AI face-swapping apps will use my data responsibly.	The degree to which users lack confidence in the responsible data handling and reliability of AI face-swapping technology and its developers.	Harrison McKnight et al., 2002; Söllner et al., 2016
	TD2 I do not trust that AI face-swapping technology can protect my personal information.		
	TD3 I feel a sense of loss of control and fear about the future development of this technology.		
Perceived enjoyment (PE)	PE1 AI face-swapping technology is very novel to me.	The degree to which users experience pleasure, fun, and intrinsic satisfaction when using AI face-swapping technology.	Voss et al., 2003; Venkatesh et al., 2003; van der Heijden, 2004
	PE2 I find experiencing AI face-swapping to be very interesting.		
	PE3 Creating face-swapped content makes me feel happy.		
Perceived usefulness (PU)	PU1 Using AI face-swapping helps me create content quickly.	The degree to which users believe that using AI face-swapping technology enhances their content creation efficiency or achieves specific functional goals.	Davis, 1989; Venkatesh and Davis, 2000
	PU2 AI face-swapping improves the quality of my social media content.		
	PU3 AI face-swapping technology is useful to me.		
Need for social identification (NSI)	NSI1 I hope my AI face-swap content gets likes.	The degree to which users are motivated by the desire to obtain social recognition, approval, and a sense of group belonging through creating and sharing AI face-swap content.	Utz et al., 2012
	NSI2 I care about how others evaluate my face-swap creations.		
	NSI3 I want my face-swapped videos to be recognized and appreciated by others.		
Digital literacy (DL)	DL1 I understand how AI face-swapping technology works.	The degree to which individuals possess the ability to critically understand how AI face-swapping technology works, identify AI-generated content, and actively verify the authenticity of such content.	Ng, 2012; Hwang et al., 2023
	DL2 I can identify AI-generated face-swap content.		
	DL3 I actively verify the authenticity of AI face-swap content.		
Self-efficacy (SE)	SE1 I am capable of using AI face-swapping technology skillfully.	The degree to which individuals believe in their capability to skillfully and independently operate AI face-swapping technology and resolve problems encountered during use.	Compeau and Higgins, 1995; Venkatesh et al., 2003
	SE2 I can use AI face-swapping even without guidance.		
	SE3 I can solve problems encountered when using AI face-swapping.		
Continuance intention (CI)	CI1 I intend to continue using AI face-swapping technology.	The degree to which users intend to continue using AI face-swapping technology in the future.	Bhattacherjee, 2001; Cheng, 2020
	**CI2** I will continue to use AI face-swapping in the future.		
	**CI3** I am more inclined to continue using AI face-swapping than to stop.		

### 3.3 Data collection

To thoroughly investigate the complex psychological mechanisms users face when engaging with AI face-swapping technology, this study adopted a specific, vivid research context rather than a broad technological concept: the “FacePlay” app, which triggered a phenomenal trend in the Chinese market, served as the core case, with adult users aged 18–45 as the primary research subjects. Launched in 2019, the FacePlay app rapidly gained popularity across social networks due to its low operational threshold, highly realistic video face-swapping effects, and rich entertaining templates (such as classic film and television character replacements), accumulating a substantial user base. Relevant data indicate that FacePlay has consistently ranked among the top image and video tool applications in terms of monthly active users (MAU), with cumulative downloads exceeding 10 million. This highly overlaps with the core user demographics of content distribution platforms such as TikTok and Xiaohongshu, making it an ideal window for observing the digital consumption and AI interaction behaviors of China's younger generation. The selection of FacePlay was primarily based on its development trajectory of “overnight fame” accompanied by “privacy controversies,” which perfectly embodies the core issues of this research.

The target population of this study was explicitly limited to users who had actual experience using the FacePlay app within the past 6 months. This screening criterion ensured that respondents answered questions about technology anxiety, usage enjoyment, and continuance intention based on genuine personal experience rather than imagination or hearsay, thereby substantially enhancing data validity and reliability.

#### 3.3.1 Sampling procedure

This study employed an online questionnaire survey for data collection. The questionnaire was designed and distributed through “Wenjuanxing” (https://www.wjx.cn), a professional online survey platform in China. Data collection occurred from July to September 2025, spanning approximately 3 months.To precisely target respondents, we adopted a strategy combining targeted snowball sampling with community recruitment. Initial seeds: First, known FacePlay users among team members and their acquaintances were identified as initial seeds. Social network diffusion: Recruitment posts containing the app name keyword were published on social platforms such as WeChat and Weibo, explicitly inviting “users who have used the FacePlay app” to participate in an academic survey about usage experience. Vertical community penetration: Targeted recruitment was conducted in online communities with relevant content discussions, including Douban groups, Zhihu topics, and Xiaohongshu.

#### 3.3.2 Quality control

To ensure questionnaire data quality, we implemented multiple screening mechanisms. Experience screening: A screening question was placed at the beginning of the questionnaire, such as “Have you used ‘FacePlay' or similar AI face-swapping apps to create videos in the past 6 months?” Respondents answering “No” were terminated from the survey. Attention check: A reverse-scored item was embedded in the questionnaire to identify and exclude carelessly completed responses. Platform deduplication: The Wenjuanxing platform function was utilized to record and exclude duplicate IP addresses. Response time: A minimum response time threshold was set to eliminate invalid questionnaires completed too quickly.

Through the above procedures, we collected 412 questionnaires in total. After rigorous screening, 351 valid questionnaires were obtained, yielding an effective response rate of 85.2%. All samples came from active users with genuine AI face-swapping app usage experience, establishing a solid foundation for subsequent data analysis. The demographic characteristics of the respondents are presented in Table 2.

**Table 2:** Descriptive statistics of respondents.

Measure	Items	Frequency (*n* = 351)	Percentage (%)
Gender	Male	138	39.3
	Female	213	60.7
Age	18–25 years	126	35.9
	26–30 years	112	31.9
	31–35 years	68	19.4
	36–45 years	45	12.8
Education level	High school or below	42	12.0
	Associate degree	79	22.5
	Bachelor's degree	172	49.0
	Master's degree or above	58	16.5
Usage experience	1–6 months	89	25.4
	7–12 months	124	35.3
	1–2 years	96	27.4
	More than 2 years	42	12
Primary usage purpose	Entertainment	187	53.3
	Short video creation	89	25.4
	Social sharing	56	16.0
	Commercial/ professional use	19	5.4

### 3.4 Data analysis method

This study employed Partial Least Squares Structural Equation Modeling (PLS-SEM) for data analysis using SmartPLS 4.0 software (Sarstedt et al., 2022). PLS-SEM was selected for the following reasons: First, the research model contains higher-order constructs (push effect and pull effect), and PLS-SEM possesses advantages in handling complex model structures (Hair et al., 2019). Second, PLS-SEM has more relaxed requirements for data distribution, not requiring multivariate normality, making it more suitable for survey research (Hair et al., 2017). Third, PLS-SEM is particularly appropriate for exploratory and predictive research; this study aims to explore the relative strength of push and pull effects and the moderating role of mooring factors, which aligns with PLS-SEM application scenarios (Sarstedt et al., 2020).

To address concerns regarding statistical power, an a-priori power analysis was conducted using G^*^Power 3.1. For a model with 12 predictors (including interaction terms, a significance level α of 0.05, and a power level 1 – β) of 0.80, the required sample size to detect a small effect size (*f*^2^ = 0.02) is approximately 340. Our actual sample size of 351 fulfills this criterion, confirming that the study is sufficiently powered to identify even subtle moderating effects. This ensures that the observed interaction effects in our model are statistically reliable and not due to insufficient sensitivity.

## 4 Results

### 4.1 Common method bias test

Since the data in this study were derived from self-reported measures, common method bias may exist. During the survey administration, the researchers emphasized the anonymity and confidentiality of the questionnaire and clarified that the data would be used solely for scientific research purposes to minimize common method bias sources.

Additionally, Harman's single-factor test was employed to examine common method bias (Hair et al., 2021). Results indicated that nine factors with eigenvalues greater than 1 were extracted without rotation, with the first factor explaining 22.51% of the variance, which is below the 40% threshold. These results suggest that no serious common method bias exists among the variables, and the findings are within acceptable limits.

### 4.2 Measurement model assessment

Following the two-stage analytical procedure recommended by Hair et al. (2019), we first evaluated the reliability and validity of the first-order reflective constructs (Table 3). All standardized factor loadings ranged from 0.700 to 0.936, exceeding the 0.70 threshold. Construct reliability was evidenced by Cronbach's alpha (α) and Composite Reliability (CR) values. For most constructs, *a* values exceeded 0.70, while Digital Literacy and Self-Efficacy demonstrated acceptable internal consistency with *a*> 0.60 in this specific context (Hair et al., 2014a,b). All CR values exceeded 0.795, and the Average Variance Extracted (AVE) for all constructs ranged from 0.565 to 0.830, consistently exceeding the 0.50 threshold, thus confirming robust convergent validity. Variance Inflation Factor (VIF) values for all indicators were below 3.0, indicating no multi-collinearity concerns.

**Table 3:** First-order reflective constructs—factor loadings and reliability.

Construct	Loading	VIF	a	CR	AVE
Need for social identification					
NSI1	0.907	2.394	0.877	0.914	0.781
NSI2	0.803	2.369			
NSI3	0.936	2.483			
Perceived enjoyment					
PE1	0.791	1.217	0.763	0.866	0.684
PE2	0.889	2.519			
PE3	0.870	2.433			
Perceived identity threat					
PIT1	0.913	2.879	0.898	0.936	0.83
PIT2	0.907	2.632			
PIT3	0.913	2.789			
Perceived privacy invasion					
PPI1	0.912	2.726	0.887	0.930	0.816
PPI2	0.898	2.572			
PPI3	0.900	2.494			
Perceived usefulness					
PU1	0.894	2.351	0.755	0.861	0.676
PU2	0.819	1.247			
PU3	0.843	2.143			
Technology distrust					
TD1	0.887	2.334	0.873	0.922	0.797
TD2	0.883	2.304			
TD3	0.908	2.524			

To provide full transparency of measurement model results at the item level, Table 4 presents the complete cross-loadings matrix for all constructs. All indicators load most strongly on their intended constructs, with cross-loadings on non-target constructs remaining substantially lower, providing item-level evidence of discriminant validity

**Table 4:** Cross-loadings matrix.

Indicator	NSI	PE	PIT	PPI	PU	TD
NSI1	**0.907**	−0.029	0.22	0.323	0.028	0.272
NSI2	**0.803**	−0.047	0.216	0.26	−0.013	0.273
NSI3	**0.936**	−0.01	0.227	0.285	0.026	0.305
PE1	−0.035	**0.791**	0.111	0.062	0.712	0.003
PE2	−0.004	**0.889**	0.063	−0.004	0.661	0.014
PE3	−0.021	**0.870**	0.012	0.036	0.612	−0.031
PIT1	0.233	0.072	**0.913**	0.281	0.067	0.272
PIT2	0.221	0.049	**0.907**	0.346	0.048	0.255
PIT3	0.217	0.082	**0.913**	0.315	0.075	0.268
PPI1	0.327	−0.005	0.317	**0.912**	0.007	0.264
PPI2	0.308	0.057	0.322	**0.898**	0.055	0.254
PPI3	0.251	0.048	0.295	**0.900**	0.049	0.281
PU1	0.001	0.695	0.057	0.057	**0.894**	0.018
PU2	0.048	0.699	0.011	−0.019	**0.819**	0.015
PU3	0.015	0.574	0.104	0.059	**0.843**	0.022
TD1	0.235	0	0.282	0.243	0.026	**0.887**
TD2	0.282	−0.015	0.214	0.253	−0.002	**0.883**
TD3	0.33	0	0.28	0.292	0.035	**0.908**

Bold values indicate loadings of items on their intended constructs.

Discriminant validity was assessed using the Fornell-Larcker criterion (Fornell and Larcker, 1981; Shuhaiber and Mashal, 2019), which requires that the square root of a construct's average variance extracted be greater than its correlations with other constructs. As shown in Table 5, all diagonal values exceed their corresponding inter-construct correlations, demonstrating good discriminant validity.

**Table 5:** Discriminant validity and correlation matrix.

Construct	CI	DL	NSI	PE	PIT	PPI	PU	SE	TD
CI	**0.899**								
DL	0.481	**0.890**							
NSI	0.293	0.260	**0.884**						
PE	0.042	−0.012	0.002	**0.827**					
PIT	0.247	0.327	0.203	0.074	**0.911**				
PPI	0.301	0.363	0.288	0.037	0.345	**0.903**			
PU	0.101	0.004	0.047	0.800	0.070	0.041	**0.822**		
SE	0.512	0.414	0.286	0.049	0.316	0.356	0.081	**0.901**	
TD	0.421	0.377	0.270	−0.005	0.291	0.295	0.023	0.344	**0.893**

CI, Continuance Intention; DL, Digital Literacy; NSI, Need for Social Identification; PE, Perceived Enjoyment; PIT, Perceived Identity Threat; PPI, Perceived Privacy Invasion; PU, Perceived Usefulness; SE, Self-Efficacy; TD, Technology Distrust. Bold diagonal values represent the square root of the Average Variance Extracted (AVE) for each construct. Off-diagonal values are inter-construct correlations.

To further strengthen construct validity evidence for the higher-order constructs, Table 6 presents the outer weights, *t*-values, and variance inflation factor values for the two second-order formative constructs—push effect and pull effect. All variance inflation factor values fell below 3.0, confirming the absence of multicollinearity concerns among the sub-constructs (Petter et al., 2007; Hair et al., 2019). Core sub-constructs demonstrated statistically significant outer weights, while non-significant sub-constructs were retained on theoretical grounds to preserve content validity, consistent with established partial least squares structural equation modeling practice (Hair et al., 2019, 2021).

**Table 6:** Second-order formative constructs—outer weights.

Construct	Sub-construct	Outer weight	*t*-value	VIF
Pull factors	NSI	0.955	15.897	1.006
	PE	−0.168	0.718	2.799
	PU	0.366	1.715	2.799
Push factors	PIT	0.183	1.458	1.188
	PPI	0.362	3.057	1.191
	TD	0.743	7.633	1.146

Finally, Table 7 presents the complete discriminant validity assessment using the Heterotrait-Monotrait ratio. All Heterotrait-Monotrait ratio values fall below the conservative threshold of 0.85, providing stringent evidence that each construct is empirically distinct from all others, jointly confirming adequate discriminant validity across all constructs in the model.

**Table 7:** Discriminant validity—Fornell-Larcker criterion and HTMT ratios.

Construct	NSI	PE	PIT	PPI	PU	TD
Heterotrait-monotrait ratio (HTMT) test results						
NSI						
PE	0.050					
PIT	0.278	0.094				
PPI	0.366	0.058	0.386			
PU	0.032	0.421	0.094	0.068		
TD	0.361	0.031	0.328	0.334	0.030	
Fornell-Larcker test results						
NSI	0.884					
PE	−0.023	0.827				
PIT	0.245	0.074	0.911			
PPI	0.327	0.037	0.345	0.903		
PU	0.025	0.800	0.07	0.041	0.822	
TD	0.317	−0.005	0.291	0.295	0.023	0.893

Bold diagonal values in the Fornell-Larcker matrix represent the square root of the Average Variance Extracted (AVE) for each construct, all of which exceed the inter-construct correlations in their respective rows and columns, confirming discriminant validity. All HTMT values fall below the conservative threshold of 0.85, confirming discriminant validity.

### 4.3 Structural model assessment

#### 4.3.1 Measurement model and construct weight analysis

As shown in Table 8, the weights of all three first-order dimensions of the Push Effect were highly significant (*p* < 0.001). Among these, perceived privacy invasion had the highest weight, followed by perceived identity threat, indicating that privacy concerns are the most critical driver in constituting user anxiety. Meanwhile, in the Pull Effect, perceived enjoyment was also a key dimension with significant weight. This analysis validated the effectiveness of the second-order construct measurement and revealed the relative importance of its internal dimensions. As shown in Table 8, all sub-construct weights reached statistical significance (^***^p < 0.001), confirming the validity of the second-order construct measurement.

**Table 8:** Analysis results of weights.

Construct	Sub-construct	Weights
Push factors	Perceived identity threat (PIT)	0.400^***^
	Perceived privacy invasion (PPI)	0.411^***^
	Technology distrust (TD)	0.293^***^
Pull factors	Perceived enjoyment (PE)	0.387^***^
	Perceived usefulness (PU)	0.383^***^
	Need for social identification (NSI)	0.364^***^

^*^p < 0.05; ^**^p < 0.01;

^***^p < 0.001.

#### 4.3.2 Structural model evaluation

**Figure 2:**
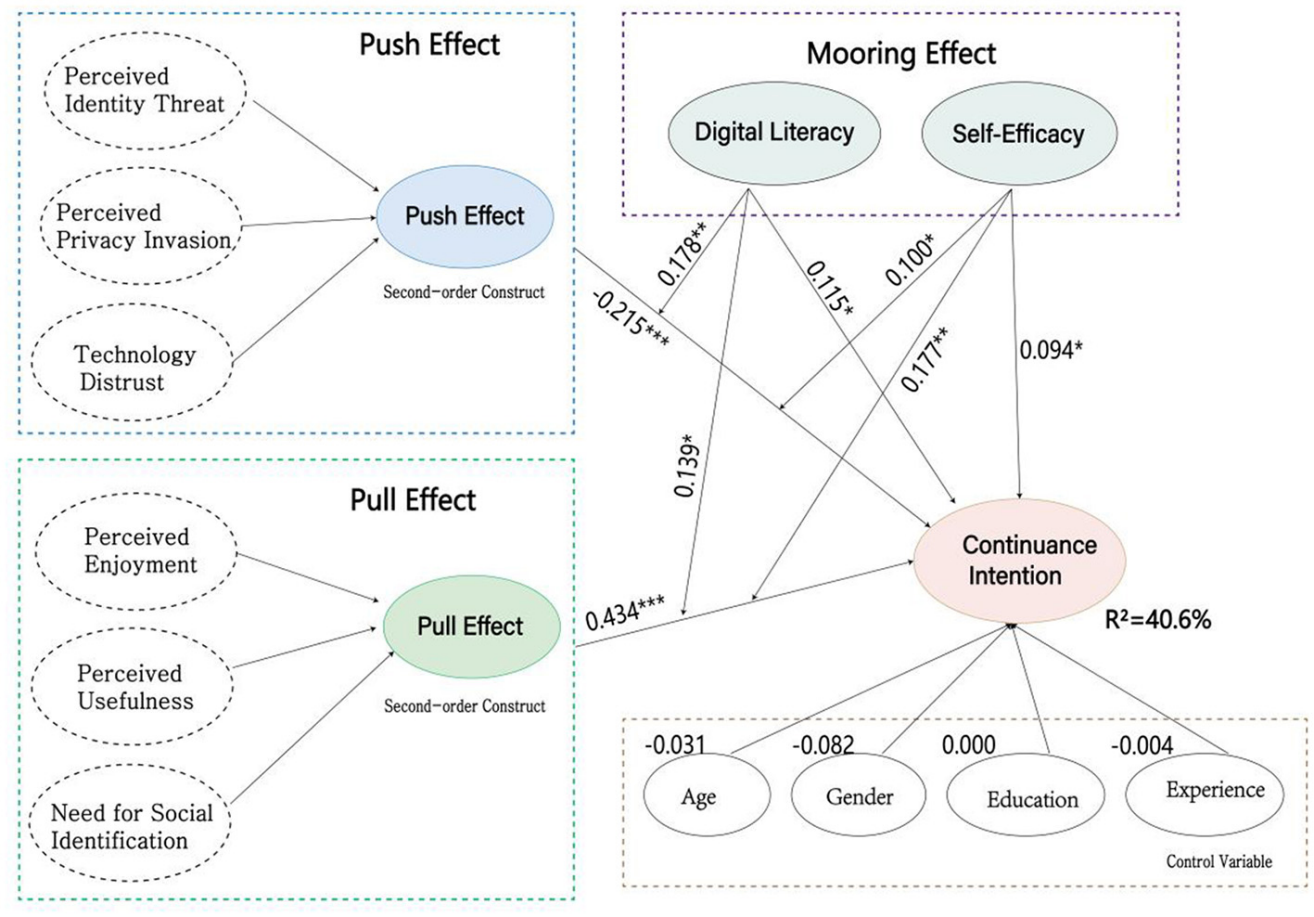
Structural model results.

In the second stage, the structural model was evaluated. We assessed the coefficient of determination (*R*^2^), predictive relevance (*Q*^2^), standardized root mean square residual (SRMR), normed fit index (NFI), and the statistical significance of path coefficients (Hair et al., 2019). *R*^2^ values between 0.33 and 0.67 indicate that the model has relatively significant explanatory power (Hair et al., 2014c). The *R*^2^ value in the model was 0.406 for continuance intention (CI). Furthermore, based on *f*^2^ values, we found that H1, H2, H3a, and H4b provided strong explanatory power for the dependent variable in the model, while other hypotheses showed weaker explanatory power (Figure 2 and Table 9). We further tested the Stone-Geisser's *Q*^2^ of the constructs using the blindfolding procedure, yielding a result of 0.353, which exceeded the recommended threshold of 0 (Hair et al., 2019). Therefore, our structural model demonstrated good fit.

**Table 9:** The results of hypothesis testing.

Hypothesis		Path coefficients	*T* values	*P* values	*F* ^2^	*R* ^2^	Result
H1	Push -> CI	−0.215	4.868	0.000^***^	0.069	40.6%	Accepted
H2	Pull-> CI	0.434	9.274	0.000^***^	0.294		Accepted
H3	DL -> CI	0.115	2.141	0.032^*^	0.024		Accepted
H4	SE -> CI	0.094	2.123	0.034^*^	0.012		Accepted
Moderating effect of digital literacy							
H3a	DL × Push -> CI	0.178	3.003	0.003^**^	0.044		Accepted
H3b	DL × Pull -> CI	0.139	2.383	0.017^*^	0.027		Accepted
Moderating effect of self-efficacy							
H4a	SE × Push -> CI	0.100	2.174	0.030^*^	0.017		Accepted
H4b	SE × Pull -> CI	0.177	3.425	0.001^**^	0.049		Accepted

^*^p < 0.05;

^**^p < 0.01;

^***^p < 0.001.

#### 4.3.3 Hypothesis testing

We validated our research hypotheses using the bootstrapping procedure in SmartPLS 4.0 with 5,000 resamples. Hypothesis significance levels were evaluated through path coefficients (β), *t*-values, and *p*-values (Hair et al., 2012; Cheung and Lau, 2008). Table 9 presents the path coefficient results. Most hypotheses in the research model were supported. The variance explained (*R*^2^) for continuance intention (CI) was 40.6%, indicating good explanatory power of the model.

##### 4.3.3.1 Direct effect of push effect on continuance intention

The push effect exhibited a significant negative influence on continuance intention (β = −0.215, *t* = 4.868, *p* < 0.001), indicating that perceived identity threat, perceived privacy invasion, and technology distrust triggered by AI face-swapping technology significantly inhibited users' continuance intention. This demonstrates that technology anxiety indeed drives users away from AI face-swapping applications, supporting the hypothesis that the push effect serves as an inhibiting factor in the research framework.

##### 4.3.3.2 Direct effect of pull effect on continuance intention

The pull effect exhibited a significant positive influence on continuance intention (β = 0.434, *t* = 9.274, *p* < 0.001), with the strongest influence strength among all direct effects. This indicates that positive factors such as perceived enjoyment, perceived usefulness, and need for social identification can effectively attract users to continue using AI face-swapping technology, supporting the hypothesis that the pull effect serves as a facilitating factor.

##### 4.3.3.3 Direct effect of mooring factors on continuance intention

Digital literacy (DL) had a significant positive influence on continuance intention (β = 0.115, *t* = 2.141 *p* < 0.05), indicating that higher digital skill levels are associated with stronger continuance intention. Self-efficacy (SE) also had a significant positive influence on continuance intention (β = 0.094, *t* = 2.123, *p* < 0.05), indicating that users' confidence in their ability to use AI face-swapping technology enhances continuance intention. These results support the hypothesis that mooring factors serve as foundational drivers.

##### 4.3.3.4 Effects of control variables

Age (β = −0.031, *p* > 0.05), gender (β = −0.082, *p* > 0.05), education level (β = −0.000, *p* > 0.05), and usage experience (β = −0.004, *p* > 0.05) showed no significant effects on continuance intention, indicating that these demographic factors did not significantly moderate user behavioral intention in the model.

#### 4.3.4 Moderation effect analysis

The moderation effect analysis focused on how DL and SE moderate the relationships between Push Effect, Pull Effect, and continuance intention. The path coefficients of interaction terms indicated that all moderating effects were significant, with specific analyses as follows:

##### 4.3.4.1 Moderating effect of digital literacy

DL exhibited a significant positive moderating effect on the relationship between push effect and continuance intention (β = 0.178, *t* = 3.003, *p* < 0.01). This result indicates that users with higher DL experience weaker negative influence of push effect on continuance intention. In other words, users with high DL can more rationally cope with technology anxiety and will not completely abandon use due to fear.

DL also exhibited a significant positive moderating effect on the relationship between pull effect and continuance intention (β = 0.139, *t* = 2.383, *p* < 0.05). This indicates that users with high DL can more fully perceive and utilize the positive value of AI face-swapping technology, thereby strengthening the positive influence of the pull effect.

##### 4.3.4.2 Moderating effect of self-efficacy

SE exhibited a significant moderating effect on the relationship between push effect and continuance intention (β = 0.100, *t* = 2.174, *p* < 0.05). This indicates that users with high SE demonstrate stronger psychological resilience when facing technology anxiety, weakening the negative influence of the push effect.

SE exhibited a significant positive moderating effect on the relationship between pull effect and continuance intention (β = 0.177, *t* = 3.425, *p* < 0.01). This indicates that when users have more confidence in their technology control abilities, the attractive effect of the pull effect is further enhanced, meaning the effect of “temptation” factors is more pronounced among users with high SE.

**Figure 3:**
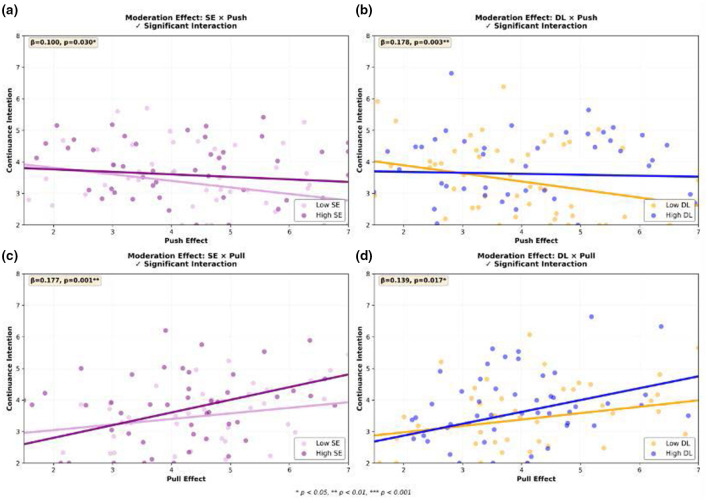
Moderation effect analysis. **(a)** Moderating effect of SE on push effect (β = 0.100, *p* < 0.05); **(b)** Moderating effect of DL on push effect (β = 0.178, *p* < 0.01); **(c)** Moderating effect of SE on pull effect (β = 0.177, *p* < 0.01); **(d)** Moderating effect of DL on pull effect (β = 0.139, *p* < 0.05). Scatter colors distinguish high/low moderator variable groups, and solid lines represent fitted regression lines. All interaction effects reached significant levels.

To further validate the moderating effects of DL and SE, this study conducted visualization analysis of the interaction effects between push-pull effects and continuance intention. Figure 3 displays the moderation effect patterns of DL and SE. From the distribution of scatter plots and regression lines, it can be clearly observed that:

For Push Effect (left two panels): For users with high SE or DL, the slopes of regression lines are more gradual. This intuitively confirms the previous conclusion that high levels of SE and DL can buffer the negative influence of anxiety factors (Push Effect) on continuance intention.

For Pull Effect (right two panels): For users with high SE or DL, the slopes of regression lines are steeper. This clearly demonstrates that high levels of SE and DL can enhance the positive influence of temptation factors (Pull Effect) on continuance intention.

## 5 Discussion

### 5.1 Push effect: the weight of fear

The significant negative influence of the push effect on continuance intention confirms the substantive inhibitory impact of the fear-driven forces collectively constituted by perceived identity threat, perceived privacy invasion, and technology distrust on user behavior, a finding consistent with prior research on the effects of technology anxiety on user behavior (Lim et al., 2025; Shen et al., 2025). Notably, among the three dimensions of the push effect, perceived privacy invasion carried the highest weight, surpassing perceived identity threat. This suggests that the leakage of facial biometric data is fundamentally irreversible: videos in which one's identity has been manipulated can theoretically be debunked, and reputational damage can to some extent be repaired, but once facial data have been collected and disseminated, they permanently escape the individual's sphere of control (Wang M. et al., 2025). This irreversibility confers upon perceived privacy invasion a form of enduring psychological pressure that places it in a dominant position within the push effect. This finding extends the conclusions of existing risk perception research (Malhotra et al., 2004), revealing the distinctive nature of privacy risk perception in the AI face-swapping context—namely, that the irreplaceable nature of facial biometric features endows perceived privacy invasion with a unique psychological impact that distinguishes it from the conclusions of general digital privacy risk research.

### 5.2 Pull effect: the power of temptation

The positive influence of the pull effect on continuance intention was substantially stronger than the negative influence of the push effect—an asymmetry that constitutes one of the most important substantive findings of this study and partially explains why AI face-swapping technology has maintained strong user engagement despite sustained risk controversy. This result is consistent with the general patterns observed in hedonic technology adoption research (Nguyen et al., 2025; Van der Heijden, 2004), indicating that in the usage decisions surrounding AI face-swapping technology, the temptation forces constituted by entertainment gratification, functional value, and need for social identification collectively outweigh the resistance generated by fear. The most recent empirical research targeting face-swapping applications has corroborated this asymmetry (Nguyen et al., 2025). This finding aligns with the core proposition of the technology paradox theory—that users do not make simple binary choices between “use” and “avoidance” but rather arrive at behavioral decisions within the sustained tension between two competing forces (Mick and Fournier, 1998)—while further demonstrating that in the specific context of AI face-swapping technology, temptation holds a relative advantage in this dynamic. This finding carries important implications for understanding the diffusion of AI entertainment technology: even when users are fully aware of technological risks, strong hedonic motivation and social drives are sufficient to sustain their continued engagement (Soto-Sanfiel et al., 2025; Li et al., 2024).

### 5.3 Mooring effect: individual capability as a boundary condition

The direct positive influence of digital literacy and self-efficacy on continuance intention indicates that individual capability conditions function not only as boundary conditions moderating push and pull dynamics, but also as independent drivers of continued engagement. This finding is consistent with prior research demonstrating that digital literacy directly enhances users' willingness to engage with new technologies by strengthening their capacity to evaluate and navigate technological environments (Getenet et al., 2024; Musyaffi et al., 2024), and that self-efficacy directly reinforces behavioral intention by bolstering users' confidence in their ability to effectively operate technology (Compeau and Higgins, 1995; Venkatesh et al., 2003; Tailor and Tailor, 2025). In the context of AI face-swapping technology, users with higher digital literacy are better equipped to assess the true value of the technology and make informed usage decisions, while users with stronger self-efficacy hold greater confidence in their ability to master its functions—both of which directly sustain continued engagement independent of push and pull forces. This finding enriches theoretical understanding of mooring variables within the Push-Pull-Mooring framework, suggesting that in AI technology contexts, individual capability conditions operate through a dual mechanism: simultaneously moderating the push-pull dynamic and independently driving behavioral intention.

Furthermore, both digital literacy and self-efficacy simultaneously moderate the strength of push and pull effects through a dual moderating mechanism: users with higher digital literacy and self-efficacy are better able to buffer the inhibitory influence of fear (push effect) on behavioral intention while simultaneously amplifying the catalytic influence of temptation (pull effect) on actual usage behavior. This dual moderating pattern—buffering resistance while amplifying motivation—reveals the complex boundary condition roles through which mooring variables operate in technology paradox contexts.

It is noteworthy that although the observed effect size of the moderating effects is relatively small (*f*^2^ = 0.017), it falls well within the anticipated range for interaction effects in social science research. According to Kenny (2016), since product terms compound measurement errors, effect sizes as low as 0.005 can still hold significant theoretical value in moderation analysis. Furthermore, Aguinis et al. (2005), in a retrospective review of 30 years of research, indicated that the average observed effect size for moderation is only approximately 0.002. Consequently, the value of 0.017 in this study is not only statistically significant but also substantially exceeds the industry average, representing a robust empirical finding.

Specifically, the modest effect sizes of digital literacy and self-efficacy offer a profound insight into the technology paradox explored in this study. These findings suggest that because the pull effects—such as perceived enjoyment and perceived usefulness—exert such a dominant influence on user behavior, individual cognitive capabilities can only play a constrained moderating role. In other words, the power of temptation inherent in these digital platforms is so potent that it partially overrides individual self-regulation. This underscores a critical narrative of our study: relying solely on individual capabilities is insufficient to mitigate the dark side of technology. Consequently, this highlights the urgent necessity for structural interventions, such as policy regulations and platform-level transparency mechanisms, to provide a more fundamental layer of protection beyond individual effort.

### 5.4 Effects of control variables

Age, gender, education level, and usage experience all showed no significant influence on continuance intention, indicating that users' continuance decisions regarding AI face-swapping technology are primarily driven by psychological perception factors rather than demographic characteristics. Regardless of age, gender, or educational background, users exhibit a high degree of consistency in their behavioral logic when confronting push and pull forces, suggesting that demographic variables do not function as boundary conditions in this decision-making process. This finding provides indirect support for the theoretical choice in this study to center psychological perception variables as the core explanatory framework.

The non-significant effect of usage experience is particularly noteworthy. In some technology adoption research, usage experience evolves over time into habit, which subsequently replaces or attenuates the influence of perception factors on behavioral intention (Venkatesh et al., 2003). However, the findings of the present study indicate that even among long-term users, continuance intention continues to be dynamically driven by push and pull forces, with psychological perception factors showing no signs of attenuation due to habituation. A plausible explanation lies in the highly dynamic nature of AI face-swapping content: each usage encounter involves new creative content and fresh social feedback, and this continuous novel stimulation suppresses the formation of habituation effects, keeping psychological perception factors actively influential throughout the entire usage cycle (Xu, 2025). This offers a proposition worthy of deeper exploration in future research: in high-dynamism content technology contexts, can habit still effectively predict continuance intention?

## 6 Conclusions and implications

This study drew on the PPM as its theoretical framework to systematically examine the mechanisms underlying CI formation among users of AI face-swapping technology from the perspective of the technology paradox. Based on empirical data from 351 active FacePlay users, the findings reveal the following core conclusions.

The push effect exerted a significant negative influence on continuance intention, with perceived privacy invasion carrying the highest weight among the three push dimensions, followed by perceived identity threat and technology distrust. This indicates that users' concerns regarding the collection and misuse of their facial biometric features constitute the most fundamental source of fear driving avoidance tendencies, and that the psychological impact of this fear derives from the irreversibility of facial data leakage. The positive influence of the pull effect on continuance intention was substantially stronger than the negative influence of the push effect; perceived enjoyment, perceived usefulness, and need for social identification collectively constitute a temptation force that outweighs fear, partially explaining why AI face-swapping technology has maintained strong user engagement despite sustained risk controversy. Digital literacy and self-efficacy not only directly and positively influence continuance intention but also simultaneously moderate the strength of both push and pull effects, manifesting a “dual moderating mechanism” whereby users with higher digital literacy and self-efficacy are both better able to buffer the inhibitory influence of fear on behavioral intention—exerting a buffering effect on the push effect—and better positioned to translate temptation into actual usage behavior—exerting an amplifying effect on the pull effect.

### 6.1 Theoretical implications

The theoretical contributions of this study operate on three levels. First, this study extends the Push-Pull-Mooring framework to the AI face-swapping technology context under the theoretical lens of the technology paradox, for the first time systematically integrating within a single model the joint influences of fear (push effects) and temptation (pull effects) on users' continuance intention. Existing research has tended to focus either on risk perceptions of AI technology or on usage motivations, with few studies examining both within a unified framework. The integrative framework developed in this study transcends this fragmentation of perspectives, providing a systematic theoretical explanation for the mechanisms underlying user behavioral decisions in technology paradox contexts.

Second, this study reveals the asymmetric nature of push-pull dynamics—the influence strength of the pull effect is significantly greater than that of the push effect. This finding provides empirical grounding for understanding the internal logic of AI entertainment technology's continued diffusion amid risk controversy, demonstrating that in the context of AI face-swapping technology—which combines strong hedonic appeal with substantial risk—users are not rational balancers but rather make continuance decisions under a temptation-dominated decision logic. This conclusion carries refinement implications for the technology paradox theory: the two poles of the paradox are not equivalent, and temptation holds a systematic advantage in particular technology contexts. Cross-cultural research has similarly found that in users' decisions to switch to AI-generated agents, the strength of emotionally capability-driven attractiveness (pull) exceeds that of functional dissatisfaction (push), underscoring the dominant role of affective design factors in AI adoption (Song and Zhou, 2026).

Third, this study identifies a dual moderating mechanism of digital literacy and self-efficacy, a finding that enriches theoretical understanding of mooring mechanisms by demonstrating that individual capability conditions do not merely constrain behavioral outcomes but actively shape the relative strength of competing motivational forces. Crucially, the relatively small effect sizes observed for these moderating effects offer a novel theoretical perspective: in the context of AI face-swapping technology, the “power of temptation” is so dominant that it partially overrides the cognitive moderation exercised by individuals through digital literacy and self-efficacy. This suggests that while digital literacy and self-efficacy constitute important boundary conditions, they do not function as absolute barriers against technological temptation. Consequently, research attention must shift from individual-level agency toward macro-level structural interventions as the primary mechanism for governing the risks associated with AI face-swapping technology.

### 6.2 Practical implications

#### 6.2.1 For platform designers

This study found that perceived privacy invasion carries the highest weight within the push effect, surpassing perceived identity threat and technology distrust (Nguyen et al., 2025), indicating that users' concerns regarding the misuse of facial data constitute the most fundamental barrier to continuance intention. Platform designers should treat privacy transparency as a foremost priority rather than an afterthought (Ali et al., 2025). Specifically, platforms can present the processes of data collection, processing, and storage in the form of visual flowcharts at the point of users' first facial image upload, clearly communicate data retention periods and the scope of third-party data sharing, and provide a one-click deletion function that users can operate independently. These measures can transform users' passive apprehension into active trust in the platform's security commitments, weakening the inhibitory influence of push effects at the source.

This study also found that the influence strength of the pull effect is substantially greater than that of the push effect, and that perceived enjoyment carries the highest weight within the pull dimension. This indicates that continuously enhancing the entertainment experience is the highest-leverage strategy for sustaining user engagement (van der Heijden, 2004; Eken, 2021). Platforms should prioritize the iterative updating of creative tools as a core product investment—for instance, by continuously expanding character template libraries, improving the real-time rendering quality of face-swapping effects, and developing social functions such as multi-person collaborative face-swapping—so that the entertainment experience generates sustained attractiveness and further reinforces the dominant position of pull forces in the push-pull dynamic.

#### 6.2.2 For differentiated user segment operations

This study found that digital literacy and self-efficacy both exert significant moderating effects on push and pull forces, indicating that a uniform platform strategy cannot effectively serve all users and that differentiated operations are indispensable (Yuan and Li, 2025).

For users with lower digital literacy, the inhibitory influence of push effects on their behavior is more pronounced. Platforms can design a “trust onboarding journey” for this user segment—embedding concise privacy protection explanations in the registration process, presenting data usage rules through accessible animation rather than legal terminology, and providing operational assistance and safety prompts during use to help users establish a basic sense of technological control (Masotina and Spagnolli, 2022). These interventions can reduce the anxiety generated by technological opacity and alleviate the inhibitory influence of push forces on continuance intention.

For users with higher digital literacy, the facilitating influence of pull effects is more pronounced. Platforms should make advanced creative functions and application programming interface access available to this segment to meet their deep usage needs, while simultaneously building a creator incentive ecosystem—for instance, through traffic support for high-quality face-swap content and community certification mechanisms—to strengthen their social identification satisfaction and further amplify the driving force of pull effects (Syed Abu Bakar et al., 2026).

#### 6.2.3 For policymakers

This study found that technology distrust is an important component dimension of the push effect, rooted in algorithmic opacity and the continued accumulation of technology misuse incidents. This finding carries direct implications for regulatory policy. China's Regulations on the Management of Deep Synthesis Internet Information Services requires prominent labeling of AI-generated content, an institutional arrangement that can help reduce users' uncertainty regarding the authenticity of technological outputs (Lumen, 2025). However, labeling requirements alone are insufficient—regulatory authorities can further promote the establishment of mandatory certification standards for facial data protection, require platforms to regularly publish data security transparency reports, and provide credible third-party endorsement of platforms' privacy commitments, thereby systematically reducing users' technology distrust at the institutional level.

Furthermore, the empirical findings of this study—particularly the dominant pull effect and the modest moderating capacity of individual capability variables—highlight a critically important policy implication: relying solely on individual digital literacy and self-regulation is insufficient to mitigate the risks associated with AI face-swapping technology. Given that the “power of temptation” can effectively bypass individual cognitive buffers, structural interventions are indispensable. Regulatory authorities should move beyond simple labeling requirements and establish mandatory “safety-by-design” standards. This includes mandatory third-party audits of biometric data processing practices and the implementation of “hard” regulatory boundaries to prevent platforms from exploiting highly addictive affective design features in the absence of adequate risk safeguards. Such structural measures provide a foundational layer of protection that individual capability alone cannot achieve.

## 7 Limitations and future research

This study has several limitations that point to important directions for future research.

First, the cross-sectional survey design captures user attitudes at a single point in time, precluding examination of how continuance intention evolves dynamically as the technology matures and regulatory environments shift. Future research should employ longitudinal tracking designs to capture the dynamic evolution of push-pull dynamics over time, and experimental designs that directly manipulate push and pull factors would enable more robust causal inference.

Second, this study focuses on continuance intention rather than actual usage behavior. Future research should incorporate objective behavioral measures—such as user log data and usage frequency statistics—to verify whether intention translates into behavior, thereby more accurately reflecting the ultimate outcomes of the fear-vs.-temptation dynamic.

Third, the sample is restricted to Chinese FacePlay users aged predominantly 18–35, recruited via snowball sampling through online communities, giving rise to two interrelated generalizability limitations. Regarding cultural specificity, the findings reflect a regulatory and cultural context shaped by China's 2022 Regulations on the Management of Deep Synthesis Internet Information Services and distinctive Chinese social media dynamics. The observed dominance of pull over push effects may be partly attributable to these context-specific factors and may not generalize to Western contexts governed by the General Data Protection Regulation or to Southeast Asian platform ecosystems. Regarding age representativeness, the predominance of younger users means that findings may not capture the technology anxiety profiles or digital capability levels of older demographic groups, for whom push effects may be considerably stronger. Additionally, snowball sampling may have introduced self-selection bias, potentially overestimating pull effect strength and underestimating push effect strength. While this does not invalidate the structural relationships identified, it calls for caution in generalizing path coefficient magnitudes. Future research should employ cross-cultural probability sampling across contrasting regulatory environments and deliberately oversample older and lower-literacy user groups to examine the boundary conditions of the Push-Pull-Mooring framework more systematically.

Looking ahead, future studies could broaden the range of mooring variables to include privacy concern disposition, personal innovativeness, and risk aversion. The fear-vs.-temptation framework could also be extended to other contested technology domains—such as facial recognition, gene editing, and autonomous driving—to test the universality of the Push-Pull-Mooring model. Longitudinal tracking of whether the relative strength of push and pull effects shifts as AI technology evolves would yield valuable insights into technology diffusion and societal acceptance processes.

## Data Availability

The original contributions presented in the study are included in the article/supplementary material, further inquiries can be directed to the corresponding author.
